# Beliefs about sleep: links with ruminations, nightmare, and anxiety

**DOI:** 10.1186/s12888-023-04672-5

**Published:** 2023-03-24

**Authors:** Julie Faccini, Vrutti Joshi, Pierluigi Graziani, Jonathan Del-Monte

**Affiliations:** LSP Laboratory, University of Nîmes, Aix-Marseille University, UR 849, 29 Avenue Robert Schuman, Aix-en-Provence, 13621 France

**Keywords:** Insomnia, Non-constructive ruminations, Emotional disorders, Sleep disorders

## Abstract

**Objective:**

Dysfunctional cognitions related to sleep play a major role in insomnia but also in nightmares. Moreover, they are closely related to anxiety. To our knowledge, no study has probed the impact of non-constructive ruminations on these dimensions in their harmful interplay with sleep. The aim of this study is to provide new insights into the processes underlying the dysfunctional cognitions-insomnia relationship.

**Method:**

Four hundred twenty nine French participants completed an anonymous online survey using Qualtrics® software. For the assessment of variables, we used the Mini Cambridge-Exeter Repetitive Thought Scale, the Nightmare Distress Questionnaire, the Beck Anxiety Inventory and the Sleep Condition Indicator. The frequency of nightmares was assessed subjectively via an item. Participants were divided into two groups according to their score on the insomnia assessment: good sleepers and poor sleepers.

**Result:**

Anxiety was found to be a common mediator of the relationship between dysfunctional beliefs and attitudes toward sleep and insomnia between good (20.8%) and poor sleepers (24.6%). However, for poor sleepers, nightmare frequency (15.2%) and non-constructive ruminations (16.6%) emerged as mediators of this relationship.

**Conclusion:**

The results gathered through this study bring interesting perspectives regarding the theoretical and etiological conceptualization of insomnia. We showed a positive association between dysfunctional beliefs and attitudes towards sleep and non-constructive ruminations in their contributory role to insomnia.

## Introduction

Dysfunctional beliefs about sleep play a significant role in several sleep disorders [1]. In Harvey’s cognitive model of insomnia [2], these beliefs explain the onset and maintenance of insomnia. Dysfunctional beliefs about sleep were found to be significantly related to several factors involved in sleep disorders such as nightmares [3–5], anxiety [6, 7] and cognitive rumination [8, 9]. Previous studies highlight the interoperability between these same factors [10, 11]. However, no study has attempted to differentiate constructive from non-constructive ruminations and assess their specific links with sleep disorders and dysfunctional thoughts and attitudes toward sleep. Watkins [12] distinguished two types of ruminations: constructive ruminations and non-constructive ruminations or repetitive negative thoughts. Constructive ruminations, defined as “*concrete and experiential thoughts*” such as “how can I get out of this?” were identified as being adaptive for individuals. In contrast, non-constructive ruminations, described as “*abstract and analytical thoughts*”, such as "why is this happening to me?”, are thought to be detrimental to one’s mental health. Few studies differentiate between types of rumination and their distinct links in relation to sleep disorders. Faccini et al. [13] have shown that only non-constructive ruminations are associated with nightmare distress, insomnia and increased psychopathological risk including suicidal risk in the general population. Tighe et al. [14] have highlighted in their work that it is important to distinguish between different types of repetitive thinking. They show that "savoring", i.e., a pattern of positive repetitive thoughts, was associated with lower levels of sleep impairment, but not with sleep disturbance in contrast to ruminations, which were significantly associated with higher levels of sleep disturbance and sleep impairment*.* To fill these gaps in the literature, it is promising to test the unexplored links between constructive and non-constructive ruminations, anxiety, nightmare distress, dysfunctional thoughts and attitudes towards sleep and insomnia in relation to the quality of sleep. We believe that a better understanding of the interlinked dynamics of these constructs may allow for a clearer comprehension of the pathway from normal sleep to insomnia. Moreover, this clarification could also provide new therapeutic targets for optimizing cognitive-behavioral and emotional therapies for insomnia. We hypothesize that only non-constructive ruminations, nightmares and anxiety are involved in the processes underlying the relationship between dysfunctional beliefs and attitudes towards sleep and insomnia.

## Method

Six hundred ninety five French participants completed an anonymous online evaluation via Qualtrics® (https://www.qualtrics.com). Participants were not compensated in any way for their participation. The questionnaire was in French only**.** Data from 429 participants were retained for statistical analysis upon excluding inappropriate responses and missing data (*n* = 266). In fact, the participants who did not fully answer the questions were excluded from the study. Responses where more than one choice was selected were deemed inappropriate and were removed from the data. The questionnaire was distributed between 29 March 2021 and 11 May 2021 on social media (Facebook, WhatsApp, Twitter) to recruit a maximum number of participants. At the very beginning of the online survey, participants were informed about the purpose of the study, the study procedures and data analysis. Participants were informed about their voluntary participation, confidentiality, objectives, methods, institutional affiliations, expected benefits and potential risks of the research and the inconvenience it may cause. Participants completed a single online self-report survey including questions related to demographic details. The total duration of the research protocol was approximately 30 min. The inclusion criteria were to be at least 18 years old and to understand, read and speak French fluently.

## Measures

The Beck Depression Inventory II (BDI-II) [15] assesses the intensity of depressive symptoms over the past two weeks. This questionnaire consists of 13 items with response options ranging from 0 to 3 points, with a maximum possible score of 39. A score > 4 indicates no depression, > 7 indicates mild depression, > 15 indicates moderate depression and > 16 indicates severe depression. The BDI-II demonstrated acceptable internal consistency (α = 0.93). We used the validated French version of this scale in this study. This scale was used only to control the clinical aspects of the samples.

The Sleep Condition Indicator (SCI) [16], an 8-item rating scale, was used to assess insomnia. Participants were asked to rate the severity of their nocturnal and daytime symptoms on 4-point Likert scales (0–3), lower scores indicate a higher insomnia intensity. The cut-off value is ≤ 16. The French version of the SCI scale showed good internal consistency (α = 0.87).

The Nightmare Distress Questionnaire (NDQ) [17] is a 13-item measure used to assess distress related to nightmares. Participants are asked to rate the items according to their frequency from never to always. Scores range from 0–52. The internal consistency is good (α = 0.88). The validated French version was used for this study.

For the assessment of anxiety, the Beck Anxiety Inventory (BAI) was used [18]. It contains 21 items that measure anxiety-related symptoms. Subjects were asked to rate the severity of their anxiety on 4- point Likert scales (0–3). It has satisfactory internal consistency (α = 0.85). We used the validated French version of this scale in this study.

The Mini Cambridge-Exeter Repetitive Thought Scale (Mini-CERTS) [19] is a 16-item questionnaire used to measure constructive and non-constructive ruminations. Items were rated on a scale of 1 to 4 (1 = almost never, 4 = almost always). Participants rated the items to reflect the way they usually think when faced with a difficult situation. In our study, the scores represent only non-constructive ruminations. The Mini-CERTS demonstrated acceptable internal consistency (α = 0.71). The validated French version was used for this study.

The Dysfunctional Beliefs and Attitudes about Sleep Scale (DBAS) [20] was used to assess dysfunctional beliefs and attitudes associated with sleep around five themes (misattribution and amplification of the consequences of insomnia, control and prediction of sleep, unrealistic expectations about sleep, preconceived notions about the causes of insomnia, false beliefs about strategies to promote sleep). The scale is organized into thirty items and responses are assessed on a ten-point Likert scale (0 to 10). A higher score indicates a higher degree of dysfunctional beliefs. The scale has an acceptable internal consistency (α = 0,79). We used the validated French version of this scale in this study.

Nightmare frequency (NF) was assessed by asking participants "In the last 3 months, on average, how often did you have nightmares? Participants could answer: no nightmares; one night/month; two nights/month; one night/week; two to three nights/week; four nights/week; five nights/week; six to seven nights/week. Responses were converted into a score from 0 to 8, with 8 representing the highest frequency.

### Statistical analysis

The statistical analyses were executed using the *Jamovi* software. Descriptive statistics were calculated for all study variables. The sample was divided into two groups: good sleepers and poor sleepers. Good sleepers refer to the participants who scored above 16 on the Sleep Condition Indicator (*N* = 223). Poor sleepers refer to the participants who scored 16 or less on the Sleep Condition Indicator (*N* = 206). Pearson correlational analyses were calculated for nightmare distress (NDQ), non-constructive ruminations (Mini-CERTS), anxiety (BAI), insomnia (SCI), dysfunctional sleep beliefs and attitudes (DBAS) and nightmare frequency (NF). Regression analysis was performed for each group. For each analysis, we tested the following variables: frequency of nightmares, nightmare distress, constructive and non-constructive ruminations, anxiety, dysfunctional beliefs and attitudes towards sleep. We have presented only the significant predictor variables. For the good sleepers’ group, the predicted variable was insomnia. The predictors were dysfunctional beliefs, constructive ruminations and attitudes towards sleep and anxiety. For the poor sleepers’ group, the predicted variable was insomnia. The predictors were dysfunctional beliefs and attitudes towards sleep, anxiety, and the nightmare frequency. For each group, we tested all the variables and retained only those that appeared significant in the regression analyses Mediation analyses were conducted for each group to better understand the relationship between dysfunctional beliefs and attitudes towards sleep and insomnia mediated by anxiety, non-constructive ruminations and nightmare frequency. All the variables were tested but only the significant ones were retained. Furthermore, in order to be able to compare the groups, we wished to present the scores of the significant mediating variables in the poor sleepers group as well as in the good sleepers group, even if they were not significant.

## Results

The demographic variables were controlled (Table 1). Descriptive statistics were used to assess the mean and standard deviation of the scores for each group: Nightmares Distress Questionnaire (NDQ) (Good sleepers: *N *= 223, M = 9.36; SD = 7.61; Poor sleepers: *N *= 206, M = 14.53; SD = 9.22), Nightmare‘s frequency (NF) (Good sleepers: *N *= 223 M = 2.04; SD = 1.36; Poor sleepers: *N *= 206, M = 3.01; SD = 1.74), Beck Depression Inventory-II (BDI) (Good sleepers: *N *= 223 M = 4.62; SD = 4.28; Poor sleepers: *N *= 206, M = 9.56; SD = 5.65), Beck Anxiety Inventory (BAI) ( Good sleepers: *N *= 223 M = 7.81; SD = 6.72; Poor sleepers: *N *= 206, M = 16.92; SD = 10.33), Sleep Condition Indicator (SCI) (Good sleepers: *N *= 223 M = 23.74; SD = 4.43; Poor sleepers: *N *= 206, M = 11.00; SD = 3.45), Cambridge-Exeter Repetitive Thought Scale (Mini-CERT) (Good sleepers: *N *= 223 M = 16.41; SD = 4.03; Poor sleepers: *N *= 206, M = 19.53; SD = 4.45) Beliefs and Attitudes about Sleep Scale (DBAS) (Good sleepers: *N *= 223 M = 103.95; SD = 37.37; Poor sleepers: *N *= 206, M = 145.70; SD = 34.68) (Table 2).

**Table 1:** Demographic characteristics for good sleepers (*N* = 223) and poor sleepers (*N* = 206)

Total Sample (*N* = 429)	Good sleepers (*n* = 223) Mean ± SD	Poor sleepers (*n* = 206) Mean ± SD
Age (years)	41.76 ± 11.86	41.08 ± 10.73
Gender (M/F)	47/223 (21.07%/78.93%)	24/182 (21.07%/78.93%)
Marital status		
Single	52 (23.4%)	53 (25.7%)
Cohabitation	91 (40.9%)	82 (39.8%)
Married	78 (34.9%)	69 (33.5%)
Other	2 (0.8%)	2 (1%)
Level of education		
(% ≥ Bachelor’s degree)	193 (86.54%)	165 (80.09%)
Professional activity		
(Yes/No)	202/223 (90.58%/9.42%)	168/206 (81.55%/18.45%)

**Table 2:** Descriptive statistics for primary study variables for the good sleepers group (*N* = 223) and the poor sleepers group (*N* = 206)

Measure	Mean		SD	
	good sleepers	poor sleepers	good sleepers	poor sleepers
Nightmare Frequency	2.04	3.01	1.36	1.74
BDI	4.62	9.56	4.28	5.65
BAI	7.81	16.92	6.72	10.33
SCI	23.74	11.00	4.43	3.45
NDQ	9.36	14.53	7.61	9.22
Non-constructive ruminations	16.41	19.53	4.03	4.45
Constructive ruminations	21.4	20	3.87	3.52
DBAS	103.95	145.70	37.37	34.68

*BDI* Beck Depression Inventory, *BAI* Beck Anxiety Inventory, *SCI* Sleep Condition Indicator, *NDQ* Nightmares Distress Questionnaire, *DBAS* Dysfunctional Beliefs and Attitudes about Sleep Scale

Correlation analyses were conducted for each group according to the quality of sleep (Tables 3 and 4). For the good sleepers’ group, anxiety, non-constructive ruminations, dysfunctional beliefs about sleep, nightmare distress and nightmare frequency were found to be significantly positively correlated with each other. Constructive ruminations were significantly negatively correlated with anxiety and dysfunctional beliefs about sleep. Constructive ruminations were found to be significantly positively correlated with insomnia. As for insomnia, the results showed a negative correlation (reverse items) of this variable with anxiety, non-constructive ruminations, dysfunctional beliefs about sleep, nightmare distress and nightmare frequency. To the correlation analyses with the poor sleepers’ group: anxiety, non-constructive ruminations, dysfunctional beliefs about sleep, nightmare distress and nightmare frequency were found to be positively correlated with each other. In relation to insomnia, the results showed a negative correlation (reverse items) with anxiety, non-constructive ruminations, dysfunctional beliefs about sleep, nightmare distress and nightmare frequency. With respect to constructive ruminations, results showed significantly positively correlations with insomnia and significant negative correlations with anxiety and dysfunctional beliefs about sleep.

**Table 3:** Correlations between clinical variables for the good sleepers

Variables	1	2	3	4	5	6	7
1. BAI	-	0.444**	0.288*	0.403**	-0.367**	0.381**	-0.136*
2. NDQ	0.625**	-	0.205*	0.420**	-0.211**	0.625**	-0.71
3. DBAS	0.288**	0.205**	-	0.156*	-0.356**	0.206**	-0.154*
4. Non-constructives ruminations	0.403**	0.420**	0.156*	-	-0.205**	0.320**	-0.019
5. SCI	-0.367**	-0.211**	-0.356**	-0.205**	-	-0.157*	0.219**
6. Nightmare Frequency	0.381**	0.625**	0.206**	0.320**	0.157*	-	0.032
7. Constructive ruminations	-0.136*	-0.71	-0.154*	-0.019	0.219**	0.032	-

*BAI* Beck Anxiety Inventory, *NDQ* Nightmares Distress Questionnaire, *DBAS* Dysfunctional Beliefs and Attitudes about Sleep Scale, *SCI* Sleep Condition Indicator

^*^p ≤ 0.05

^**^p ≤ 0.01

**Table 4:** Correlation between clinical variables for poor sleepers

Variables	1	2	3	4	5	6	7
1. BAI	-	0.401**	0.390*	0.430**	-0.307**	0.315**	-0.136*
2. NDQ	0.401**	-	0.339**	0.322**	-0.228**	0.633**	0.071
3. DBAS	0.390**	0.339**	-	0.347**	-0.332**	0.255**	-0.154*
4. Mini-CERTS	0.430**	0.322**	0.347*	-	-0.253**	0.294**	-0.019
5. SCI	-0.307**	-0.228**	-0.332**	-0.253**	-	-0.269**	0.219*
6. Nightmare Frequency	0.315**	0.315**	0.255**	0.294**	-0.269*	-	-0.032
7. Constructive-Ruminations	-0136*	0.071	-0.154*	-0.019	0.219*	-0.032	-

*BAI* Beck Anxiety Inventory, *NDQ* Nightmares Distress Questionnaire, *DBAS* Dysfunctional Beliefs and Attitudes about Sleep Scale, *SCI* Sleep Condition Indicator. *NF* Nightmare Frequency

^*^p ≤ 0.05

^**^p ≤ 0.01

We performed a linear regression for both the groups.

For the good sleepers’ group, the overall regression was significant (*R*^*2*^ = 0.216, *p* < 0.01) (Table 5). Dysfunctional beliefs and attitudes towards sleep (*β* = -0.251, *p* < 0.01), constructive ruminations (*β* = 0.142, *p*. = 0.016) and anxiety (*β* = -0.282, p < 0.01) were found to be significant explanatory variables of insomnia).

**Table 5:** Predicting SCI total score from clinical variables (BAI, DBAS, constructive ruminations) for the good sleepers group

Predictors	*Adjusted R*^*2*^	*Statistics*	Bêta	*p*-value	VIF	Tolerance	S.E	95% C.I. Lower / Upper
DBAS		t = -4.179	-0.251	< 0.001	1.32	0.759	-0.008	-0.82 / -0.49
BAI		t = -4.697	-0.282	< 0.001	1.64	0.609	0.0395	0.285/ 0.130
Constructive ruminations		T = 2.438	0.142	0.016	1.03	0.967	0.071	0.33/0.314
Model	0.216	*F* = *13.202*		< 0.001	-	-	-	-

*R*^*2*^ R square, *F* Fisher, *t* t de student, *S.E* Standardized estimate, *C.I*. Confidence interval *DBAS* Dysfunctional beliefs and attitudes about sleep Scale, *BAI* Beck Anxiety Inventory

For the group of poor sleepers, the overall regression was also significant (*R*^*2*^ = 0.157, *p* < 0.01) (Table 6). Dysfunctional beliefs and attitudes towards sleep (*β* = -0.226, *p*. = 0.002), anxiety (*β* = -0.169, *p* = 0.019) and nightmare frequency (*β* = -0.158; p. = 0.02) were found to be significant explanatory variables of insomnia.

**Table 6:** Predicting SCI total score from clinical variables (BAI, Mini-CERTS, NDQ and DBAS) for the poor sleepers group

Predictors	*Adjusted R*^*2*^	*Statistics*	Bêta	*p*-value	VIF	Tolerance	S.E	95% C.I. Lower / Upper
DBAS		t = -3.20	-0.226	0.002	1.21	0.828	0.007	-0.03 / -0.008
BAI		t = -2.36	-0.169	0.019	1.25	0.798	0.02	0.10/ 0.009
Nightmare Frequency		t = -2.31	-0.158	0.02	1.14	0.880	0.13	-0.58/-0.04
Model	0.157	*F* = *13.7*		< 0.001	-	-	-	-

*R*^*2*^ R square, *F* Fisher, *t* t de student, *S.E*, Standardized estimate, *C.I*. Confidence interval, *DBAS* Dysfunctional beliefs and attitudes about sleep Scale, *BAI* Beck Anxiety Inventory

**Fig. 1:**
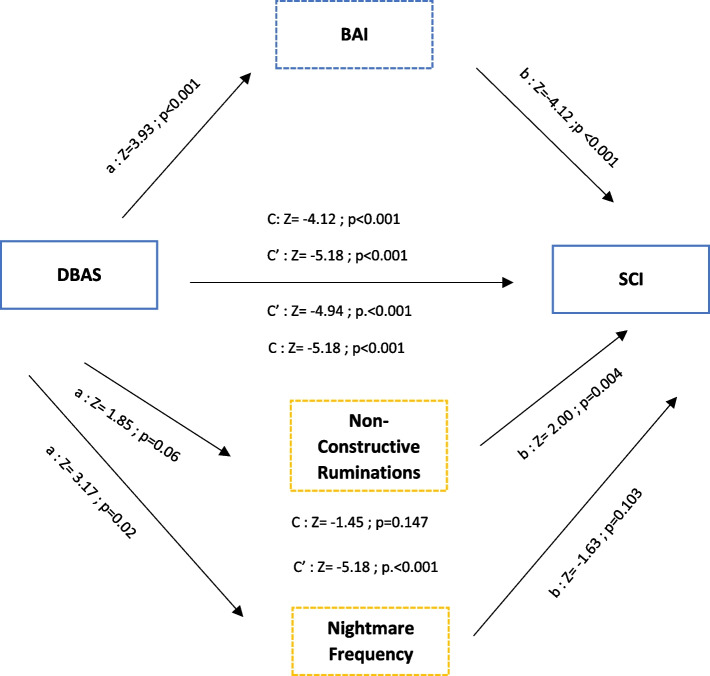
Mediation effect of dysfunctional beliefs and attitudes about sleep and insomnia for good sleepers group C = Direct effect; C’= Total effect;  = Mediation effect of the variable confirmed by Sobel test;  = Mediation not significant ; DBAS = Dysfunctional beliefs and attitudes about sleep Scale ; BAI = Beck Anxiety Inventory; SCI = Sleep Condition Indicator

**Fig. 2:**
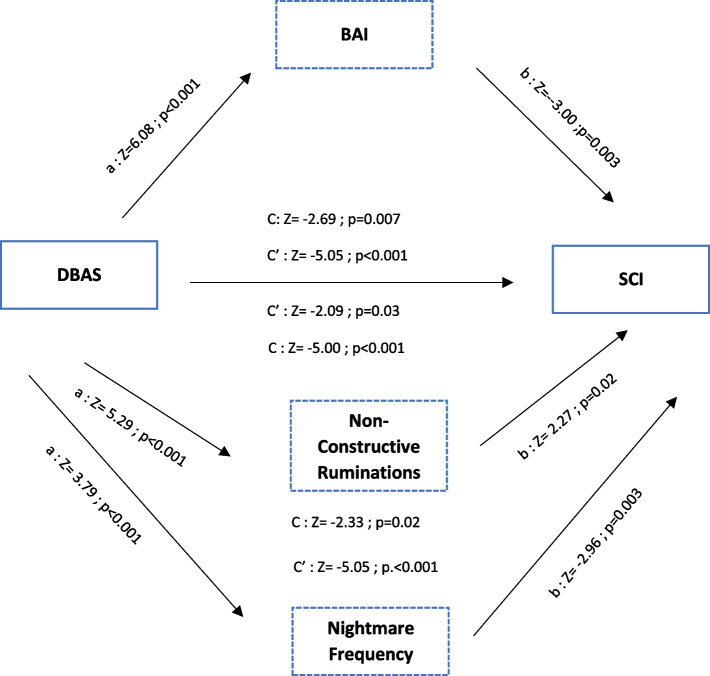
Mediation effect of dysfunctional beliefs and attitudes about sleep and insomnia for poor sleepers group C = Direct effect ; C’= Total effect;  = Mediation effect of the variable confirmed by Sobel test; DBAS = Dysfunctional beliefs and attitudes about sleep Scale ; BAI = Beck Anxiety Inventory; SCI = Sleep Condition Indicator

The results of the mediation analyses for the relationship between dysfunctional beliefs and attitudes towards sleep and insomnia are summarized in Figs. 1 and 2. Different mediation analyses were performed according to the quality of sleep.

For good sleepers, the total effect of dysfunctional beliefs and attitudes towards sleep on insomnia was significant. The mediation analysis indicated that all total indirect effects and all specific indirect effects were significant. The dysfunctional beliefs and attitudes score showed a significant indirect effect on the insomnia score mediated by anxiety (estimate = -0.008, C.I. 95% [-0.013/ -0.002], *Z* = -2.89 *p*. = 0.004) with an indirect effect of 20.8%. The Sobel test confirmed a mediating effect of non-constructive ruminations (*Z* = -102.79; *p* < 0.001).

As for the mediation analyses among the scores of poor sleepers, the dysfunctional beliefs about sleep score showed a significant effect on the insomnia mediated by the anxiety (Estimate = -0.008, C.I. 95% [-0.014/ -0.002], *Z* = -2.69, *p* = 0.007) with an indirect effect of 24.6%. The Sobel test confirmed the mediator effect (*Z* = -145.63, *p* < 0.001). A significant mediator effect of the non-constructive ruminations (Estimate = -0.005, C.I. 95% [-0.01/ -3.37], *Z* = -2.09, *p* = 0.03) with an indirect effect of 16.6% was equally found. The Sobel test confirmed the mediator effect (*Z* = -42.74, *p* < 0.001). Furthermore, we observed a significant mediator effect of the nightmare frequency (Estimate = -0.005, C.I. 95% [-0.009/ -8.02], *Z* = -2.33, *p* = 0.02) with an indirect effect of 15.2%. The Sobel test confirmed the mediator effect (*Z* = -22.4, *p* < 0.001).

## Discussion

The aim of this study was to understand the links between non-constructive ruminations, dysfunctional thoughts and attitudes towards sleep, nightmare distress, nightmare frequency and insomnia in the general population. The role of constructive ruminations was also assessed. A large number of participants met the criteria for insomnia which was evaluated using the Sleep Condition Indicator. Indeed, this observation highlighted the relevance of the purpose of our study, which was to dissect the interoperability of the variables assessed.

First, dysfunctional beliefs and attitudes towards sleep (DBAS) were positively correlated with non-constructive ruminations as well as with anxiety, nightmare distress and nightmare frequency in both groups (Tables 3 and 4). Dysfunctional beliefs and attitudes towards sleep, anxiety, nightmare distress, non-constructive ruminations and nightmare frequency were found to be negatively correlated with insomnia (reverse items). These correlation analyses were conducted for good sleepers and poor sleepers respectively. The results showed homogeneity between the two groups andare consistent with the existing scientific literature [8, 13]. In addition, constructive ruminations were negatively associated with dysfunctional beliefs about sleep and anxiety. They were, however, positively associated with insomnia (reversed items) in both groups.

In the aforementioned studies, the deleterious interoperability of insomnia-specific rumination and non-constructive ruminations along with emotional processes and nightmares is highlighted. The cognitive constructs appear to be related to sleep-related difficulties, emotional dysregulation, and anxiety. Moreover, we can observe that the scores of these variables were more strongly expressed in the group of poor sleepers. The results concerning constructive ruminations in relation to dysfunctional beliefs about sleep are very interesting. They show the importance of distinguishing the nature of ruminations, which could be favorable to the reduction of insomnia, through its cognitive determinants. These results are in line with Tighe et al. [14] study which also distinguished different types of repetitive thoughts and showed a distinction in their pathogenic function.

In our linear regression models, the two groups manifested distinct results. For the good sleepers’ group, anxiety, constructive ruminations and dysfunctional beliefs and attitudes towards sleep were significant. In the poor sleepers, anxiety, dysfunctional beliefs about sleep and frequency of nightmares were found to be significant. These results can be explained by the fact that among good sleepers, the insomnia score to be predicted is not pathological. Thus, anxiety, which is a commonly experienced emotion, and DBAS, may explain a sleep dysfunction, a tendency, but is not the only pathway for the development of insomnia. Furthermore, we can observe that the beta of constructive ruminations is positive. This can be explained by the adaptive nature of constructive ruminations which themselves serve as an efficient emotional regulation strategy that could be a protective factor against insomnia. On the other hand, in the poor sleepers’ group, in addition to the anxiety and dysfunctional beliefs and attitudes, we obtained a significant association with the frequency of nightmares. These results are in line with the literature that documents a higher frequency of nightmares as an explanatory factor of insomnia severity [21, 22]. In addition, we observed a higher occurrence of nightmares among the poor sleepers which may explain an effect of this variable on insomnia only for this group (Tables 5 and 6).

Mediation analyses were conducted to further explore the processes underlying the relationship between insomnia and DBAS. For good sleepers, we found a mediating effect of anxiety only. These results demonstrate, in line with the literature [23], that emotional processes are constantly linked to sleep, independently of the pathological aspect of the latter. In addition, the role of emotional regulation processes and most notably cognitive constructs can be evoked. A lower score of non-constructive ruminations was observed in good sleepers. Anxiety appears to mediate the DBAS-insomnia link in the group characterized by good sleep. The probable involvement of cognitive management of emotions as a protective factor against sleep disorders may explain these results. For poor sleepers, mediation analyses allowed us to demonstrate a mediating effect of anxiety, non-constructive ruminations and nightmare frequency. The interpretation of these results supports our aforementioned explanatory hypotheses. Indeed, in this group, non-constructive ruminations, constituting an abstract, non-problem-focused rumination style, are more prevalent than among the good sleepers. This type of cognition confirms their potentially detrimental impact on sleep which may also be related to dysfunctional beliefs and attitudes towards sleep. DBAS are non-resolving thoughts and attitudes, closely related to anxiety. The presence of non-constructive ruminations appears to be a real obstacle to their suppression and resolution, which would explain their interfering effect on insomnia. In addition, research has shown that sleep deprivation inhibits the suppression of unwanted thoughts [24], suggesting a perpetuating cycle of insomnia through non-constructive ruminations, DBAS and anxiety.

This work provides significant exploratory evidence regarding the role of nightmares, anxiety and non-constructive ruminations as an underlying process in the DBAS-insomnia relationship. In addition, it provides new data on the role of constructive ruminations in the interrelations between the emotional determinants of the hypnotic sphere. The interrelation of these variables is confirmed by our statistical analyses. We believe that non-constructive ruminations, considered as active agents of psychological decline [12], act as a maintenance factor for dysfunctional sleep beliefs and attitudes in poor sleepers. The results of the correlation analyses concerning constructive ruminations could express a possible protective role of these cognitions.

Future research should continue to explore these links in order to reach a consensus on their effective involvement in insomnia. It would be particularly interesting to investigate the role of these variables in the clinical population.

Our study has some limitations. Indeed, we have a non-homogeneous male–female ratio. Moreover, our work was undertaken in a non-clinical population. These factors do not allow us to generalize our results but we can however direct the path to be further explored and confirmed by future research. In addition, the online survey and social network distribution method we used is also a limitation. These methods of administration and distribution did not allow us to control for possible contextual effects. Moreover, this limits access to the questionnaires only to people who are active on social networks.

In conclusion, we believe that these results may allow an adjustment in cognitive-behavioral therapies for insomnia. By targeting non-constructive ruminations and nightmares in the treatment of dysfunctional cognitions, clinicians could potentially maximize therapeutic benefits.

## Data Availability

The datasets generated and/or analysed during the current study are not publicly available due to informed consent not including data sharing but are available from the corresponding author on reasonable request.
